# Isolation, Identification, and Molecular Characterization of *Mycoplasma bovis* from Beef Cattle in Kunming, and Development of a SYBR Green qPCR Assay

**DOI:** 10.3390/pathogens15020162

**Published:** 2026-02-02

**Authors:** Guojun Wang, Yuqing Li, Lixian Liu, Ling Zhao, Veerasak Punyapornwithaya, Wentao Zhao, Yan Liu, Tianlong Qi, Wengui Li

**Affiliations:** 1 Yunnan Joint International R&D Center of Veterinary Public Health, College of Veterinary Medicine, Yunnan Agricultural University, Kunming 650201, China; guojun.w@kkumail.com (G.W.); zwt13766321@sina.com (W.Z.); 18468111403ly@sina.com (Y.L.); 2 Faculty of Veterinary Medicine, Khon Kaen University, Khon Kaen 40002, Thailand; 3 College of Animal Science and Veterinary Medicine, Shenyang Agricultural University, Shenyang 110866, China; lyq18288696821@163.com; 4 Academy of Science and Technology, Chuxiong Normal University, Chuxiong 675000, China; liulixian@cxtc.edu.cn; 5 Kunming Institute of Zoology, Chinese Academy of Sciences, Kunming 650201, China; zhaoling@mail.kiz.ac.cn; 6 Faculty of Veterinary Medicine, Chiang Mai University, Chiang Mai 50100, Thailand; veerasak.p@cmu.ac.th

**Keywords:** *Mycoplasma bovis*, isolation and identification, antimicrobial resistance, SYBR Green I qPCR

## Abstract

*Mycoplasma bovis* (*M. bovis*) is a major pathogen responsible for bovine respiratory disease, mastitis, and arthritis, causing significant economic losses to the cattle industry worldwide. To elucidate the genetic and biological characteristics of *M. bovis* circulating in Yunnan Province, China, twenty PCR-positive bovine respiratory samples were collected from cattle farms in Kunming; three isolates—M.bo-YNXD-1, A1, and A8—were successfully cultured and identified through colony morphology, biochemical assays, and molecular characterization. Antimicrobial susceptibility testing showed that M.bo-YNXD-1 exhibited multidrug resistance to six antibiotics, including ciprofloxacin and lincomycin, while A1 and A8 were resistant to one or two agents, respectively. Multilocus sequence typing (MLST) analysis revealed that isolates M.bo-YNXD-1 and M.bo-YNXD-A8 belonged to sequence type ST52, whereas isolate M.bo-YNXD-A1 was assigned to ST90, indicating the coexistence of distinct genetic lineages in this region. Virulence gene screening showed that isolate M.bo-YNXD-A8 was positive for *VspX* and *p81*, whereas all three isolates were positive for *p48* and *Vpam*. A SYBR Green I-based quantitative PCR (qPCR) assay targeting the *oppD/F* gene was established, exhibiting high specificity, a detection limit of 10 copies/μL, and intra-/inter-assay variation below 3%. Validation using clinical samples demonstrated superior sensitivity compared with conventional PCR. Taken together, these findings indicate the presence of distinct MLST genotypes and virulence-associated genetic heterogeneity among regional *Mycoplasma bovis* isolates, and introduce a rapid, sensitive, and reliable qPCR assay for early detection and epidemiological surveillance. This study provides critical insights for rational antimicrobial use and targeted control strategies against *M. bovis* infections.

## 1. Introduction

*Mycoplasma bovis* (*M. bovis*) is a principal pathogen responsible for the bovine respiratory disease complex (BRDC), a multifactorial syndrome involving multiple viral and bacterial agents, including Mannheimia haemolytica, Pasteurella multocida, Histophilus somni, Mycoplasma dispar, bovine viral diarrhea virus (BVDV), bovine herpesvirus type 1 (BoHV-1), bovine respiratory syncytial virus (BRSV), and parainfluenza virus type 3 (BPIV-3), which together cause substantial economic losses to the global cattle industry [1,2]. Beyond respiratory disease in calves, *M. bovis* is also associated with arthritis, mastitis, and otitis media, frequently resulting in chronic and refractory infections that are difficult to eradicate [1,2]. The absence of a cell wall renders *M. bovis* intrinsically resistant to β-lactam antibiotics, while its repertoire of variable surface proteins (Vsps) enables antigenic variation, immune evasion, and long-term persistence within the host [3,4]. These biological features, combined with its ability to form biofilms and modulate host immune responses, make *M. bovis* one of the most challenging pathogens to control in modern cattle production systems.

In China, the rapid intensification of the cattle industry and frequent interregional animal movement have accelerated the spread of *M. bovis*, which is now recognized as a major bacterial pathogen of economic and veterinary importance [5]. The overly frequent indiscriminate use of antimicrobials for control of the widespread spread has further driven the emergence of multidrug-resistant (MDR) *M. bovis* strains. Increasing resistance to macrolides, fluoroquinolones, and lincosamides—such as tylosin, enrofloxacin, and lincomycin—has been reported in multiple regions, complicating therapeutic options and contributing to treatment failures [6]. Serological surveys have demonstrated extensive *M. bovis* exposure across China, with antibody prevalence rates of 31.99% in Xinjiang and 24.90% in Ningxia, and regional variation ranging from 27.1% in the southwest to 38.7% in the northwest [7]. These findings highlight the pathogen’s widespread distribution and regional epidemiological heterogeneity.

Yunnan Province represents the key area of China’s beef cattle production. According to the Yunnan Bureau of Statistics (2023), Yunnan ranks first nationwide in cattle inventory (8.97 million head) and second in slaughter volume (3.65 million head), producing 447,400 tons of beef annually https://stats.yn.gov.cn/Pages_23_3934.aspx (accessed on 20 September 2025). Despite this strong industrial presence, comprehensive investigations into the genetic diversity, virulence gene profiles, and antimicrobial resistance characteristics of *M. bovis* in Yunnan remain limited. The lack of regional data hinders the establishment of region-specific control measures.

Accurate and rapid diagnosis is a key issue to effective *M. bovis* control; however, current diagnostic approaches have notable limitations. Although culture-based isolation and identification are considered the gold standard, they are laborious, time-consuming, and often yield low recovery rates due to the organism’s fastidious growth requirements [8]. Conventional PCR assays offer faster detection but lack the sensitivity and quantification capability required for clinical and epidemiological applications. Quantitative PCR (qPCR) has been widely adopted for *M. bovis* detection [9,10]; yet most existing assays were designed based on reference strains from other regions and may not fully capture the genetic variability of emerging field strains in southwestern China. To address these limitations, the oligopeptide permease system genes *oppD/F*, which encode ATP-binding components of an ABC transporter essential for nutrient uptake and host adaptation in *M. bovis*, were selected as the target for assay development. Previous comparative genomic studies have demonstrated that the *oppD/F* genes are highly conserved among *M. bovis* strains while exhibiting sufficient sequence divergence from other closely related Mycoplasma species, supporting their suitability as species-specific molecular targets [11]. Given the ongoing evolution of *M. bovis* and the escalating issue of antimicrobial resistance, there is an urgent need for regionally optimized molecular diagnostic tools and continuous genomic surveillance [12].

This study aimed to isolate and characterize *Mycoplasma bovis* from PCR-positive cattle samples in Kunming, Yunnan Province, China, and to analyze their genetic diversity, and virulence gene profiles. Additionally, a SYBR Green I-based qPCR assay targeting the *oppD/F* gene was developed and evaluated for the detection of *M. bovis*.

## 2. Materials and Methods

### 2.1. Sample Collection and Processing

A total of 58 clinical samples, including 56 nasal swabs and 2 lung tissue samples, suspected of *Mycoplasma bovis* infection were collected from five intensive cattle farms in Kunming, Yunnan Province, during routine veterinary diagnosis of naturally infected cattle. Samples were transported on ice and stored at −20 °C until further analysis. Nasal swabs were immersed in 2.5 mL of PPLO medium (Qingdao Haibo Biotechnology Co., Ltd., Qingdao, China) and incubated at 37 °C in a 5% CO_2_ atmosphere for 2–5 days. A color change in the medium from red to yellow was considered indicative of presumptive *Mycoplasma* growth. Cultures were centrifuged at 2500× *g* for 10 min, and the supernatants were filtered through 0.45 μm membrane filters for downstream analysis. Lung tissue samples (~0.5 cm^3^) were obtained from cattle that died naturally or were euthanized for clinical reasons unrelated to this study, homogenized in PPLO medium, and processed using the same procedure. To improve detection efficiency and reduce unnecessary isolation attempts, genomic DNA was extracted directly from processed samples and initially screened by PCR targeting the *uvrC* gene for the detection of *Mycoplasma bovis*. All samples were collected during routine veterinary diagnostic procedures from naturally infected cattle, and no experimental manipulation of live animals was performed. Therefore, ethical approval for animal experimentation was not required in accordance with institutional and national guidelines.

### 2.2. Isolation and Identification of Mycoplasma bovis

Samples that tested positive for *Mycoplasma bovis* by *uvrC* gene PCR screening were selected for isolation and further characterization. Filtered samples (0.3 mL) were inoculated into PPLO medium and incubated at 37 °C for 3–4 days. DNA extracted from cultures showing presumptive *Mycoplasma* growth was subjected to PCR targeting the *uvrC* gene to confirm the identity of *M. bovis*. PCR-confirmed isolates were subcultured three consecutive times for purification and subsequently streaked onto PPLO agar. Colonies exhibiting typical “fried-egg” morphology were selected for further purification. Microscopic features were examined by Gram, Dienes, and Giemsa staining, and ultrastructural morphology was observed by transmission electron microscopy (TEM) following negative staining.

### 2.3. Biochemical Characterization

Biochemical properties, including glucose fermentation, gelatin hydrolysis, arginine hydrolysis, mannitol utilization, and cholesterol requirement, were evaluated using a commercial identification kit according to the manufacturer’s instructions.

### 2.4. Growth Curve Determination

Purified isolates were cultured in PPLO medium at 37 °C for 5 days. Medium pH, color change, and color change units (CCU) were recorded at 12–24 h intervals. Growth curves were generated to compare proliferation characteristics among isolates.

### 2.5. PCR Detection of Mycoplasma bovis

PCR assays were performed targeting the *16S rRNA* and *uvrC* genes of *M. bovis*. For the *16S rRNA* gene, primers were FWD: ACGCGTCGACAGAGTTTGATCCTGGCT and REV: CGCGGATCCGCTACCTTGTTACGACTT, producing a 1500 bp fragment. The PCR conditions were: initial denaturation at 94 °C for 7 min, followed by 35 cycles of 94 °C for 1 min, 66 °C for 45 s, and 72 °C for 1 min, with a final extension at 72 °C for 8 min [13]. For the *M. bovis*-specific *uvrC* gene, primers were FWD: TTACGCAAGAGAATGCTTCA and REV: TAGGAAAGCACCCTATTGAT, yielding a 1626 bp amplicon. The PCR protocol consisted of an initial denaturation at 94 °C for 7 min, followed by 35 cycles of 94 °C for 1 min, 51 °C for 45 s, and 72 °C for 1 min, with a final extension at 72 °C for 8 min [14]. PCR products were analyzed by 1% agarose gel electrophoresis, and the target bands were excised, purified, and sequenced. Sequence alignments and identity analyses were performed using the online NCBI BLAST tool and MEGA 7.0, and phylogenetic trees were constructed using the Neighbor-Joining (NJ) method. Information on the reference strains used in the phylogenetic analysis is provided in Table 1.

### 2.6. Multilocus Sequence Typing (MLST)

MLST was conducted using the PubMLST *M. bovis* scheme. Seven housekeeping genes (*recA*, *gyrB*, *dnaE*, *dtdS*, *pntA*, *pyrC*, and *tnaA*) were amplified using published primers (Table S2) [15]. Purified amplicons were sequenced and assigned allele numbers and sequence types (STs) via the PubMLST database.

### 2.7. Antimicrobial Susceptibility Testing

Antimicrobial susceptibility was assessed using the agar diffusion method following CLSI guidelines (M100-33rd edition) (Table S1). Purified cultures (0.2 mL) were spread onto PPLO agar plates, and 14 antimicrobial agents were tested. Inhibition zone diameters were measured after incubation at 37 °C for 3–5 days and interpreted as susceptible, intermediate, or resistant.

### 2.8. Detection of Virulence Genes

Six primers specific to virulence genes (*VspX*, *p48*, *p81*, *Vpma*, *VspHB0801-1*, *VspY2*) were used (Table S3) [16]. PCR products were sequenced and compared with NCBI reference sequences. Multiple sequence alignments were performed using DNASTAR 7.1, and phylogenetic analysis was conducted using MEGA 11.0 with 1000 bootstrap replicates.

### 2.9. Development of SYBR Green I qPCR

A SYBR Green qPCR assay targeting the *oppD/F* gene was established using genomic DNA extracted from purified isolates. Primer design and specificity evaluation were performed using Primer Premier 5.0 and in silico analysis with NCBI Primer-BLAST against the nucleotide (nt) database, confirming the absence of predicted amplification in non-target species (Supplementary Figure S1). The primer consisted of a forward primer (5′-TCGCTTATCTCGGCTATACCT-3′) and a reverse primer (5′-CGTTGCTGCTTTGTGATGAC-3′), generating an amplicon of 186 bp. Reaction components and cycling conditions were optimized to determine amplification efficiency, standard curves, specificity, sensitivity (limit of detection), and repeatability. The assay was further validated using clinical samples. Detailed optimization procedures are provided in Supplementary Section S2.9.

## 3. Results

### 3.1. Isolation and Purification of Mycoplasma bovis

**Figure 1:**
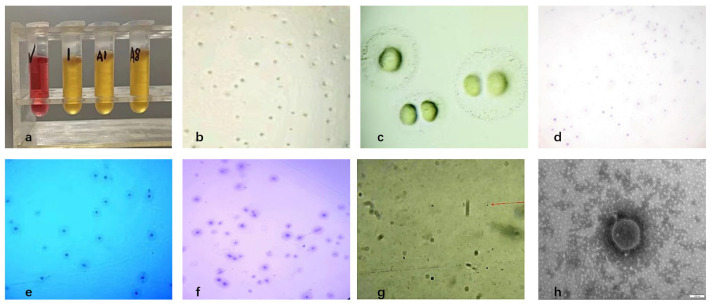
Isolation and morphological characterization of *Mycoplasma bovis*; (**a**) Growth observation in liquid culture medium; (**b**) Results of solid medium (×100); (**c**) Results of solid medium (×400); (**d**) Gram-staining results of colonies (×100); (**e**) Dienes-staining results of colonies (×100); (**f**) Giemsa-staining results of colonies (×100); (**g**) Giemsa-staining results of bacteria (×1000), as indicated by the red arrow; (**h**) Electron microscope observation of *Mycoplasma bovis* isolated (×150,000).

Three pure *Mycoplasma bovis* isolates were obtained from 21 PCR-positive bovine respiratory samples, yielding an isolation rate of 15% (Figure 1a). After three rounds of purification, colonies on PPLO agar exhibited the typical “fried-egg” appearance, characterized by a dense, raised central core and a surrounding translucent peripheral zone (Figure 1b,c). Gram staining showed weak and inconclusive staining, consistent with the lack of a peptidoglycan cell wall in *Mycoplasma*, which prevents stable retention of the crystal violet–iodine complex. (Figure 1d). Dienes staining revealed a characteristic biphasic pattern with a dark blue central region and a lighter peripheral halo (Figure 1e). Giemsa staining showed a dense dark-blue central area that gradually transitioned to a lighter periphery, with cells appearing as irregular granular or coccoid forms (Figure 1f,g). Transmission electron microscopy further confirmed typical *Mycoplasma* morphology, including pleomorphic spherical, dumbbell-like, and filamentous structures (0.1–0.3 μm), all surrounded by a trilaminar membrane and lacking a cell wall (Figure 1h). Based on colony morphology, staining characteristics, and ultrastructural features, the isolates were identified as *Mycoplasma*.

### 3.2. Identification and Phylogenetic Analysis

#### 3.2.1. PCR Validation of Isolates

**Figure 2:**
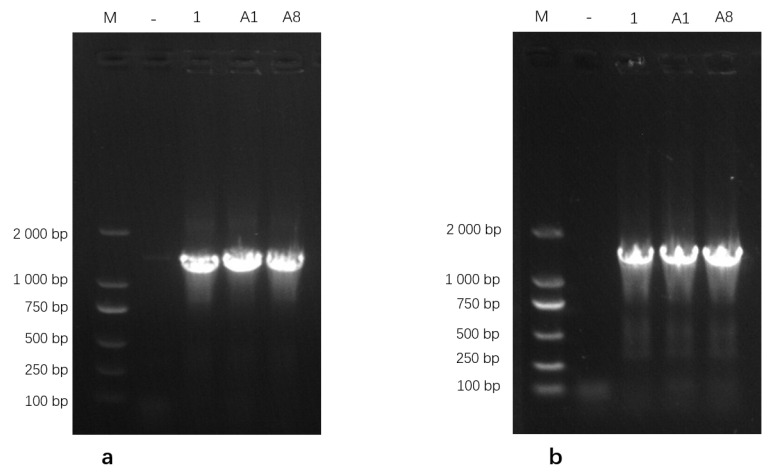
Amplification of *16S rRNA* (**a**) and *uvrC* (**b**)-specific gene from *Mycoplasma bovis* isolates.“−” indicates the negative control.

Genomic DNA extracted from the three isolates was amplified using PCR and subsequently analyzed on a 1% agarose gel. The *16S rRNA* gene-specific primers generated clear and distinct amplicons of the expected size, while amplification with the *uvrC* gene-specific primers produced a single band corresponding to the anticipated target fragment (Figure 2). These PCR amplification profiles were consistent with the expected band sizes for *Mycoplasma bovis*, further supporting the successful molecular identification of the isolates. Based on these results, the isolates were identified as *M. bovis* and designated as M.bo-YNXD-1, M.bo-YNXD-A1, and M.bo-YNXD-A8.

#### 3.2.2. Phylogenetic and Sequence Identity Analysis

**Figure 3:**
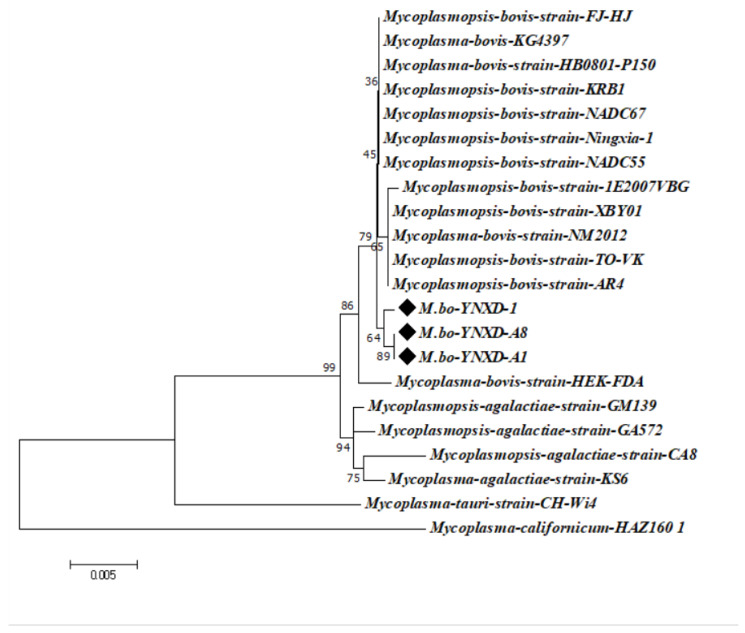
Phylogenetic tree of *Mycoplasma bovis* strains based on the *16S rRNA*. The tree was constructed using the neighbor-joining method with the Kimura 2-parameter model in MEGA version 7.0. Bootstrap analysis was performed with 1000 replicates, and bootstrap values ≥ 50% are shown at the corresponding nodes. The scale bar represents the number of nucleotide substitutions per site.“◆” indicates the isolates obtained in this study.

The *16S rRNA* gene sequences of the three isolates showed high intraspecific similarity, with pairwise identity values ranging from 99.4% to 100%. Comparable identity levels were observed with *M. bovis* reference strains from Inner Mongolia and Henan Province (99.7–99.8%) and with international epidemic strains from North America and Europe (e.g., GenBank CP016546.1; 99.4–99.8%). In contrast, identity values with other *Mycoplasmal* species were notably lower, including 98.9–99.3% for M. agalactiae, 97.0% for M. bovigenitalium, and 94.1% for M. californicum. All non–*M. bovis* comparisons remained below the 97% interspecies threshold, supporting the classification of the isolates as *M. bovis*. Phylogenetic analysis using the Neighbor-Joining method further confirmed these findings: strains M.bo-YNXD-1, M.bo-YNXD-A1, and M.bo-YNXD-A8 clustered tightly within Clade I, while reference strains 1E2007VBG (Clade II) and FJ-HJ (Clade III) formed related subgroups. Together, these clusters constituted a well-supported monophyletic group, indicating a close evolutionary relationship among the isolates and representative *M. bovis* strains (Figure 3).

### 3.3. Growth Characteristics and pH Changes of Mycoplasma bovis Isolates

**Figure 4:**
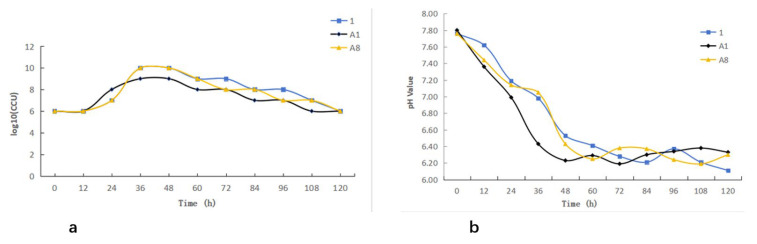
Growth kinetics of *Mycoplasma bovis*: (**a**) Growth curve of *Mycoplasma bovis*; (**b**) pH dynamic monitoring.

The three *M. bovis* isolates exhibited comparable growth characteristics. All isolates entered the logarithmic phase approximately 12 h after inoculation and reached the stationary phase at around 36 h, followed by a transition into the decline phase after 48 h (Figure 4a). Monitoring of the culture medium showed a gradual decrease in pH concurrent with bacterial growth, demonstrating an inverse relationship between biomass accumulation and pH. Notably, the onset of the decline phase corresponded with a reduction in pH to approximately 6.4 (Figure 4b).

### 3.4. Biochemical Characterization of Mycoplasma bovis Isolates

Biochemical characterization revealed that all three isolates were negative for glucose fermentation, gelatin hydrolysis, arginine hydrolysis, and mannitol utilization. In contrast, all isolates showed a positive reaction in the cholesterol growth-requirement assay. This metabolic profile, defined by cholesterol dependence and the absence of fermentative activity, is consistent with the established biochemical characteristics of *Mycoplasma bovis* (Table 2).

### 3.5. Multilocus Sequence Typing of Mycoplasma bovis Isolates

Multilocus sequence typing (MLST) was used to determine the genetic profiles of the three *M. bovis* isolates. The results showed that isolates M.bo-YNXD-1 and M.bo-YNXD-A8 were assigned to sequence type ST52, with an allele profile of dnaA-5, tkt-4, gltX-3, gpsA-2, gyrB-3, pta2-5, and tdk-3. In contrast, isolate M.bo-YNXD-A1 belonged to ST90, characterized by the allele profile dnaA-5, tkt-4, gltX-2, gpsA-3, gyrB-3, pta2-5, and tdk-7 (Table 2). ST52 has been reported as one of the predominant genotypes circulating in China and is also a major subtype identified in Australia, China, and Israel. Notably, all *M. bovis* isolates reported from Australia to date belong to ST52.

### 3.6. Antimicrobial Susceptibility Profiles of Mycoplasma bovis Isolates

Antimicrobial susceptibility testing revealed clear differences among the three *M. bovis* isolates. M.bo-YNXD-1 exhibited the broadest resistance profile, showing complete resistance to ciprofloxacin, lincomycin, and six additional antimicrobials. These resistances spanned more than three distinct antimicrobial classes, meeting the criteria for multidrug resistance (MDR). This isolate displayed intermediate susceptibility to erythromycin and enrofloxacinand remained susceptible to six agents, including norfloxacin and streptomycin. In contrast, M.bo-YNXD-A1 showed intermediate susceptibility only to erythromycinand was susceptible to the remaining 12 antimicrobials. Corresponding to resistance in fewer than three antimicrobial classes and therefore not classified as MDR. Similarly, M.bo-YNXD-A8 was resistant to erythromycin while remaining susceptible to the other 12 agents, and did not meet the MDR definition (Table 3).

### 3.7. Detection of Virulence Gene

#### 3.7.1. Virulence Gene Detection

**Figure 5:**
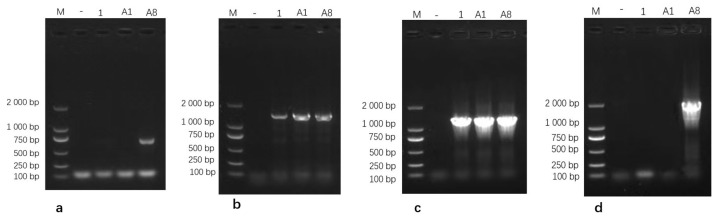
Gel electrophoresis results of *VspX* (**a**), *p48* (**b**), *Vpam* (**c**), and *p81* (**d**) Gene amplification. Note: M: DL 2000 Marker; “−” represents the negative control.

Virulence gene screening showed that isolate M.bo-YNXD-A8 was positive for *VspX* and *p81*. All three isolates were positive for *p48* and *Vpam*, but negative for *vspY2* and *vspHB0801-1* (Figure 5).

#### 3.7.2. Identity and Phylogenetic Analysis of Virulence-Associated Genes

Identity and phylogenetic analyses were conducted on four virulence-associated genes (*VspX*, *p48*, *Vpam*, and *p81*) to characterize the three *M. bovis* isolates (M.bo-YNXD-1, M.bo-YNXD-A1, and M.bo-YNXD-A8). Among these genes, *VspX* and *p81* were detected only in M.bo-YNXD-A8, whereas *p48* and *Vpam* were present in all isolates.

For *VspX*, isolate M.bo-YNXD-A8 showed 100% identity with eight domestic strains (e.g., CQ-W70, HB0801, NM2012), 96.7% identity with strains JF4278 and J279, and markedly lower identity (79.5–82.3%) with selected international strains (Figure S2). The *p48* gene was completely conserved among the three isolates, exhibiting 100% similarity with CQ-W70 and KRB1, 99.8% similarity with Xinjiang, 99.5–99.9% similarity with other *M. bovis* reference strains, and only 81.2% similarity with M. agalactiae PG2, indicating strong species specificity (Figure S3). The *Vpam* gene displayed 97.6–99.0% identity among the three isolates and 97.8–99.6% identity with domestic strains (CQ-W70, HB0801, 16M), as well as 96.7–99.6% identity with international strains (AR3-1, Mb287, Millmer) (Figure S4). The *p81* gene of M.bo-YNXD-A8 shared 98.1–98.9% identity with domestic strains (CQ-W70, HB0801, 16M, XBY01, NM2012, NX114, GuangXi, GJ2F0) and 94.1–98.9% identity with international strains (KRB1, MJ2, PG45.9, RM16, TO-VK, VK8) (Figure S5).

**Figure 6:**
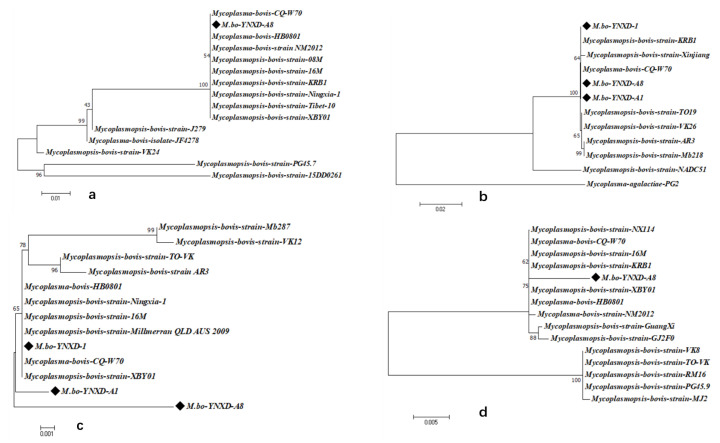
Phylogenetic analysis of virulence genes nucleotide sequences; (**a**) Phylogenetic analysis of *VspX* nucleotide sequences; (**b**) Phylogenetic analysis of p48 nucleotide sequences; (**c**) Phylogenetic analysis of Vpam nucleotide sequences; (**d**) Phylogenetic analysis of p81 nucleotide sequences. These trees were constructed using the neighbor-joining method with the Kimura 2-parameter model in MEGA version 7.0. Bootstrap analysis was performed with 1000 replicates, and bootstrap values ≥ 50% are shown at the corresponding nodes. The scale bar represents the number of nucleotide substitutions per site.“◆” indicates the isolates obtained in this study.

Phylogenetic analyses demonstrated that all Yunnan isolates clustered closely with domestic strains across the four virulence-associated genes. *VspX* and *p48* formed well-supported monophyletic clusters with Chinese isolates, indicating stable regional lineage patterns. In the *Vpam*-based tree, M.bo-YNXD-A1 and M.bo-YNXD-A8 grouped into separate branches, whereas M.bo-YNXD-1 clustered with domestic strains alongside the international Millmer strain, suggesting moderate intraspecies divergence. The *p81* gene of M.bo-YNXD-A8 clustered with domestic isolates and KRB1, whereas Guangxi and several international strains formed distinct subclades, reflecting broader global diversity. Overall, *p48* and *Vpam* were conserved among all Yunnan isolates, whereas *VspX* and *p81*—present only in M.bo-YNXD-A8—displayed high identity with domestic isolates but greater divergence from international strains, indicating regional lineage stability accompanied by global genetic variability (Figure 6a–d).

### 3.8. Establishment of SYBR Green I qPCR

#### 3.8.1. Primer Specificity

**Figure 7:**
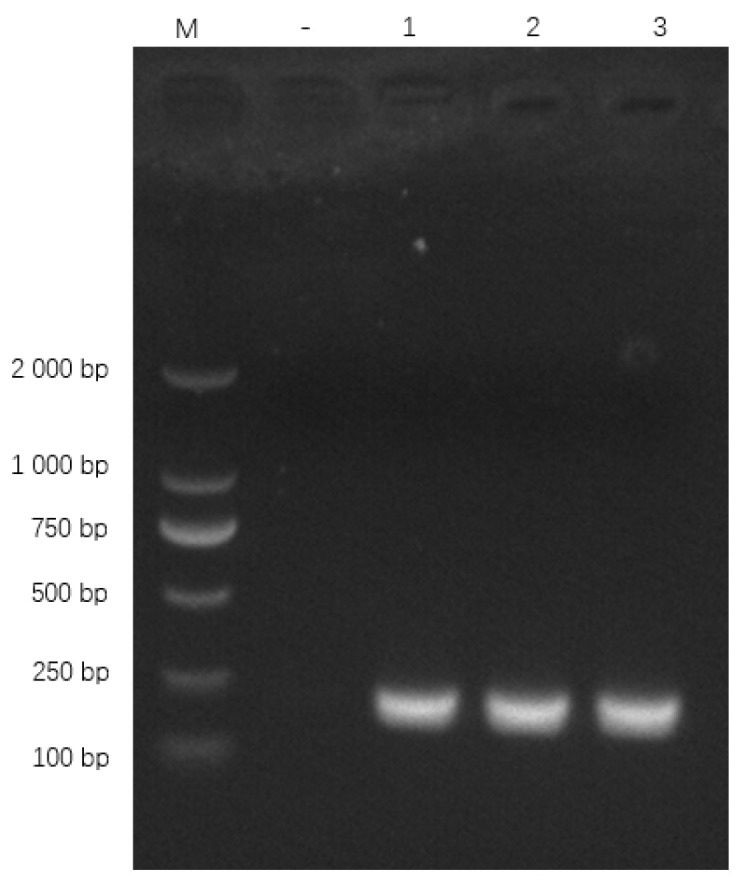
qPCR primer amplification results. Note: M: DL 2000 Marker; “−” represents the negative control, “1”, “2” and “3” represent the recombinant plasmid.

Primers specifically amplified the expected 186 bp *oppD/F* fragment (Figure 7). BLAST confirmed 100% identity with *M. bovis oppD/F* (GenBank AF130119.1).

#### 3.8.2. Optimisation of qPCR

Key parameters of the SYBR Green-based qPCR assay, including primer concentration, annealing temperature, and cycling conditions, were optimised to achieve efficient amplification of the 186 bp oppD/F fragment of *Mycoplasma bovis*. The final 20 μL reaction mixture consisted of 10 μL of 2× SYBR Green Pro Taq HS Premix II (High Rox Plus, Hunan Accurate Biotechnology Co., Ltd., Changsha, China), 0.4 μL each of the forward and reverse primers (10 μM), 2 μL of the positive plasmid template, and RNase-free water to a final volume of 20 μL. The optimised thermal cycling program included an initial denaturation at 95 °C for 30 s, followed by 40 cycles of denaturation at 95 °C for 5 s and annealing/extension at 60 °C for 30 s.

#### 3.8.3. Standard and Melt Curve

The standard curve generated using 10^9^ to 10^1^ copies/μL of the positive plasmid demonstrated a strong linear correlation between the logarithmic template concentration and Ct values (y = −3.5877× + 39.859, R^2^ = 0.9971, E = 90.0%) (Figure S6). The melt curve analysis showed a single, sharp peak at approximately 81.39 °C (Figure S7), indicating specific amplification of the target fragment, and no signal was detected in the negative control.

#### 3.8.4. Specificity Test

The specificity of the SYBR Green I qPCR assay was assessed using nucleic acids extracted from seven common microorganisms. No amplification was detected from *Mycoplasma* ovine pneumoniae, Salmonella, Providencia, Acinetobacter, Escherichia coli, Lactobacillus, or Enterococcus faecalis, whereas a clear amplification signal was obtained only from *Mycoplasma bovis* DNA (Figure S8). These findings demonstrate that the established assay is highly specific for *M. bovis* and shows no cross-reactivity with non-target microorganisms.

#### 3.8.5. Sensitivity Test

The analytical sensitivity of the SYBR Green I-based qPCR assay was evaluated to determine its limit of detection (LOD) using 10-fold serial dilutions of the recombinant pMD19-T-MBO standard plasmid, ranging from 1 × 10^9^ to 1 × 10^1^ copies/μL. Each dilution produced a typical sigmoidal amplification curve with consistent fluorescence kinetics and clear separation between adjacent dilution points, while no amplification signal was observed in the negative control. The assay consistently detected plasmid concentrations as low as 10 copies/μL, which was therefore defined as the LOD under the experimental conditions used. These results demonstrate that the established qPCR assay exhibits high analytical sensitivity and is suitable for the detection of low-copy-number *Mycoplasma bovis* DNA in clinical samples.

#### 3.8.6. Reproducibility Test

Reproducibility was assessed using standard plasmid dilutions ranging from 1 × 10^9^ to 1 × 10^5^ copies/μL. Each concentration was tested in triplicate to determine intra-assay and inter-assay variability. The intra-assay coefficient of variation (CV) ranged from 0.45% to 1.40%, and the inter-assay CV ranged from 0.50% to 2.71%. All CV values were below 3.0%, confirming that the assay exhibits excellent repeatability and stability (Table 4).

#### 3.8.7. Clinical Sample Testing

A total of 58 clinical samples were tested using the established SYBR Green I qPCR assay and a conventional PCR method (*uvrC* gene). The SYBR Green I qPCR assay identified three additional positive samples compared with conventional PCR (Table 5). These results indicate that the developed assay provides higher analytical sensitivity and improves the detection rate of *Mycoplasma bovis* in clinical specimens.

## 4. Discussion

*Mycoplasma bovis* is an obligate parasitic pathogen distinguished by the lack of a cell wall, which renders it inherently resistant to β-lactam antibiotics and poses immense challenges to clinical therapy [17]. The rapid emergence of antimicrobial resistance (AMR) and the frequent occurrence of mixed infections in bovine respiratory disease complex (BRDC) further complicate effective disease management [18,19]. As a major etiological agent of BRDC, *M. bovis*-associated pneumonia has been reported to cause an incidence rate of 50–100% in cattle herds across China, with an average mortality of approximately 10%, and over 50% during severe outbreaks [20,21]. Disease occurrence is strongly influenced by transportation stress, immunosuppression, and co-infections with other bacterial or viral pathogens [20,22]. Culturing in China displays genetic diversity yet shares clonal relationships with strains from other regions, suggesting regional dissemination associated with cattle movement [7]. Continuous monitoring of genetic and antimicrobial profiles is therefore essential to inform targeted prevention and therapeutic strategies.

Despite the significant economic impact of *Mycoplasma bovis* infections on the cattle industry, molecular epidemiological and pathogenic data on strains circulating in Yunnan Province remain scarce. In this study, three *M. bovis* strains (M.bo-YNXD-1, M.bo-YNXD-A1, and M.bo-YNXD-A8) were successfully isolated from 21 PCR-positive clinical samples collected from cattle exhibiting respiratory symptoms in the Kunming region. The isolates were comprehensively characterized using colony morphology observation, biochemical profiling—including glucose non-fermentation and cholesterol dependence—and molecular identification via *16S rRNA* sequencing. Sequence alignment revealed 99.4–100% identity with *M. bovis* reference strains, confirming accurate species identification. These findings provide foundational data for understanding the genetic diversity and pathogenic potential of *M. bovis* in Yunnan, and establish a basis for subsequent studies on antimicrobial resistance, virulence determinants, and molecular surveillance within the region.

The biochemical traits of the Kunming isolates—glucose non-fermentation and obligate cholesterol requirement—were consistent with the well-documented phenotypic characteristics of *M. bovis* [23,24]. Molecular validation further corroborated species identity: *16S rRNA* and *uvrC* genes, two widely accepted markers for *Mycoplasma* identification [6,25], shared 99.4–100% sequence identity with reference strains. Multi-locus sequence typing (MLST) analysis showed that the three isolates belonged to sequence types (STs) previously reported for *M. bovis* in China. This finding is consistent with earlier reports indicating that *M. bovis* populations in China exhibit relatively low genetic diversity, with ST10 and ST52 being among the predominant genotypes [7,25,26]. Notably, ST52 has been identified as a major subtype not only in China but also in Australia and Israel, and all *M. bovis* isolates reported from Australia to date belong to ST52, highlighting the wide geographic dissemination and epidemiological significance of this lineage.

Antimicrobial susceptibility testing uncovered substantial heterogeneity in resistance phenotypes among the three isolates. M.bo-YNXD-1 displayed resistance to six antimicrobials, including ciprofloxacin (fluoroquinolone) and lincomycin (lincosamide), while M.bo-YNXD-A1 and M.bo-YNXD-A8 showed resistance to only one or two agents. Notably, the Kunming isolates exhibited significantly higher fluoroquinolone resistance rates compared to North American *M. bovis* strains. This regional difference is likely attributed to mutations in the quinolone resistance-determining region (QRDR) of the *gyrA* gene, a well-characterised mechanism of fluoroquinolone resistance in *Mycoplasma* [27,28]. Intra-regional resistance variability (M.bo-YNXD-1 vs. M.bo-YNXD-A1/A8) was presumably driven by distinct antimicrobial usage practices: prolonged high-dose administration of quinolones and lincosamides in the farm of M.bo-YNXD-1 may have exerted strong selective pressure for resistance gene accumulation, whereas prudent antimicrobial use in the other farms reduced such pressure. Inter-regional comparisons revealed that Kunming isolates had significantly lower resistance rates to tetracyclines and macrolides (<30%) than isolates from northern China (Inner Mongolia and Heilongjiang), where resistance rates exceed 70% [9,21,29]. These discrepancies are likely associated with differences in farming systems and antimicrobial usage patterns: southern China primarily adopts extensive grazing systems with lower cattle density, while northern regions rely on intensive farming with higher disease pressure and more frequent antimicrobial application.

Host cell adhesion is a pivotal initial step in *M. bovis* pathogenesis, and adhesins are recognized as core virulence determinants [29]. Genotypic profiling of the Kunming isolates identified multiple adhesion-related virulence genes, indicating strong pathogenic potential. The variable surface protein (Vsp) family—including subtypes A, B, C, E, F, O, X, and L—was detected in all isolates. Vsp proteins not only mediate host cell adhesion but also enable immune evasion through high-frequency antigenic variation, a key strategy for *M. bovis* persistence in the host [30,31]. Additionally, membrane lipoproteins such as *p48* have been identified in all isolates, and *P48* has been reported to induce host cell apoptosis and cytotoxicity in vitro by activating endoplasmic reticulum stress-dependent apoptotic pathways, indicating a role in enhancing pathogenic effects of *M. bovis* infection [32]. Similarly, *M. bovis* infection has been shown to activate host apoptotic caspases and disrupt apoptotic regulation in macrophages, emphasizing the contribution of lipoproteins to apoptosis-related virulence phenotypes [33]. Notably, *p48*, VpmA, and *p81* genes exhibited high sequence stability across the three isolates, supporting their potential as molecular markers for diagnostic development and virulence assessment.

A SYBR Green-based quantitative PCR (qPCR) assay targeting the *oppD/F* gene was established for rapid and specific *M. bovis* detection. Compared with traditional serological methods (e.g., ELISA), this qPCR assay reduces detection time from 24 to 48 h to 2 h and avoids false negatives caused by delayed or weak host immune responses [34]. Compared with conventional PCR targeting the *16S rRNA* gene, the *oppD/F* gene offers superior species specificity. *16S rRNA* shares up to 99.47% identity with *M. agalactiae*, leading to cross-reactivity, while *oppD/F* exhibits only ~86% sequence identity with *M. agalactiae* [11,34]. The developed assay demonstrated excellent performance, high specificity against the tested non-target bacterial species, high sensitivity (limit of detection: 10 copies/µL), and strong reproducibility (intra- and inter-assay coefficient of variation < 3%). Clinical validation showed that the qPCR assay had a 15% higher detection rate than conventional PCR, making it a reliable tool for *M. bovis* diagnosis and BRDC molecular epidemiological studies.

Despite these promising results, several limitations should be acknowledged. First, the relatively small sample size and geographically concentrated sampling may not fully represent the genetic diversity and antimicrobial resistance patterns of *M. bovis* circulating in the Kunming region, potentially introducing sampling bias. Moreover, the limited inclusion of reference strains from other geographic regions may restrict the extrapolation of the phylogenetic and diagnostic findings to *M. bovis* populations outside China. Second, the pathogenic potential of the isolates was inferred primarily from genotypic and resistance analyses without in vivo validation. The absence of animal challenge experiments limits our ability to establish a direct relationship between virulence, thereby restricting a comprehensive evaluation of strain pathogenicity. These limitations may affect the generalizability of the findings, highlighting the need for broader, multi-regional studies and functional assays in future research.

## 5. Conclusions

This study offers an integrated genetic, phenotypic, and diagnostic assessment of *Mycoplasma bovis* circulating in Yunnan Province, revealing pronounced genomic variability and distinct antimicrobial resistance patterns among regional isolates. Importantly, the SYBR Green I-based qPCR assay established in this work provides a highly sensitive, specific, and reproducible tool for early detection, achieving superior diagnostic performance compared with conventional PCR. Together, these findings refine the current understanding of *M. bovis* epidemiology and resistance evolution in southern China and deliver a robust molecular diagnostic platform that will support evidence-based therapeutic decision-making and strengthen disease surveillance efforts in the cattle industry.

## Figures and Tables

**Table 1:** Reference strains used in this study.

Species	Strain Name	Accession No.	Origin (Region, Country)	Year
*Mycoplasmopsis bovis*	HB0801-P115	CP007589.1	Hubei, China	2012
	Ningxia-1	CP023663.1	Ningxia, China	2013
	XBY01	CP045797.1	Henan, China	2019
	CQ-W70	CP005933.1	Chongqing, China	2009
	Tibet-10	CP062195.1	Tibet, China	2019
	NM2012	CP011348.1	Inner Mongolia, China	2012
	16M	CP038861.1	Shandong, China	2016
	FJ-HJ	KX230478.1	Fujian, China	2015
	08M	CP019639.1	Jiangxi, China	2008
	PG45	NR_114848.1	USA	2014
*Mycoplasmopsis agalactiae*	PG2	NR_044667.2	USA	2011
*Mycoplasmopsis primatum*	HRC292	NR_025068.1	Sweden	2000
*Mycoplasma tauri*	Zaradi2	NR_181865.1	Austria	2008
*Mycoplasmopsis californica*	strain ST-6	NR_029166.1	USA	2009
*Mycoplasmopsis gallinacea*	DD	NR_025913.1	USA	2008
*Mycoplasmopsis pulmonis*	NBRC 14896	NR_113692.1	Japan	2011

**Table 2:** Allelic profiles of the *Mycoplasma bovis* isolates.

Strain	Allele Information							ST
	*dnaA*	*tkt*	*gltX*	*gpsA*	*gyrB*	*pta2*	*tdk*	
M.bo-YNXD-1	5	4	3	2	3	5	3	ST52
M.bo-YNXD-A1	5	4	3	2	3	5	7	ST90
M.bo-YNXD-A8	5	4	3	2	3	5	3	ST52

**Table 3:** In vitro antibiotic susceptibility results of *Mycoplasma bovis* isolates.

Drug Class	Drug Name	Inhibition Zone Diameter (mm)		
		M.bo-YNXD-1	M.bo-YNXD-A1	M.bo-YNXD-A8
Macrolides	Erythromycin (E15)	14 (I)	21 (I)	11 (R)
Quinolones	Ciprofloxacin (CIP)	21 (R)	33 (S)	29 (S)
	Enrofloxacin (ENR)	19 (I)	35 (S)	32 (S)
	Norfloxacin (NOR)	18.5 (S)	19 (S)	20 (S)
Lincosamides	Lincomycin (LC)	1 (R)	25 (S)	20 (S)
	Clindamycin (CC)	0 (R)	28 (S)	25 (S)
Tetracyclines	Doxycycline (DX)	10 (R)	30 (S)	30 (S)
	Tetracycline (TE)	8 (R)	28 (S)	21 (S)
Aminoglycosides	Streptomycin (SM)	16 (S)	25 (S)	26 (S)
	Gentamicin (GM)	21 (S)	26 (S)	22 (S)
	Spectinomycin (SPT)	22 (S)	41 (S)	42 (S)
	Amikacin (AK)	20 (S)	29 (S)	25 (S)
Nitrofurans	Nitrofurantoin (FN)	20 (S)	40 (S)	35 (S)

Sensitive (S), Moderately Sensitive (I), Resistant (R).

**Table 4:** qPCR reproducibility test results.

Concentration of Template (Copies/μL)	Intra-Assay Variability (n = 3)	Intra-Assay Variability		Inter-Assay Variability (n = 3)	Inter-Assay Variability	
		Ct Value (x ± SD)	CV (%)		Ct Value (x ± SD)	CV (%)
1 × 10^9^	3	7.78 ± 0.11	1.40	3	7.74 ± 0.21	2.71
1 × 10^8^	3	10.85 ± 0.06	0.55	3	11.02 ± 0.23	2.08
1 × 10^7^	3	14.05 ± 0.12	0.85	3	14.13 ± 0.07	0.50
1 × 10^6^	3	18.99 ± 0.13	0.68	3	18.55 ± 0.44	2.37
1 × 10^5^	3	22.31 ± 0.10	0.45	3	22.27 ± 0.19	0.85

**Table 5:** Comparison of clinical sample detection results between conventional PCR and SYBR Green I qPCR.

Sampling Time	Number of Samples (n)	Conventional PCR (Positive/Total)	SYBR Green qPCR (Positive/Total)
July 2023	27	5/27	7/27
December 2023	1	0	1/1
February 2024	1	0	0
March 2024	22	14/22	13/22
April 2024	2	2/2	2/2
June 2024	5	0	0
Total (n)	58	21/58	23/58
Detection rate (%)		36.20%	39.66%

## Data Availability

The data presented in the study are available from the corresponding author upon reasonable request.

## Supplementary Materials

The following supporting information can be downloaded at: https://www.mdpi.com/article/10.3390/pathogens15020162/s1, Figure S1: In silico specificity analysis of the *oppD/F* primers using NCBI Primer-BLAST; Figure S2: identity analysis of *VspX*nucleotide sequences; Figure S3: identity analysis of *p48* nucleotide sequences; Figure S4: identity analysis of *Vpam* nucleotide sequences; Figure S5: identity analysis of *p81* nucleotide sequences; Figure S6: Standard curve of the qPCR assay generated using serial dilutions of the plasmid standard; Figure S7: Melting curve analysis showing a single peak, indicating high specificity of the qPCR amplification; Figure S8: Specificity test results of the assay; Table S1: Interpretation criteria for antimicrobial susceptibility of *Mycoplasma bovis*; Table S2: Primers information for MLST housekeeping genes; Table S3: Primers for detection of virulence genes; S2.9: Establishment and validation of the qPCR assay.

## Abbreviations

The following abbreviations are used in this manuscript:

*M. bovis*	*Mycoplasma bovis*
Vsps	Variable surface proteins
PPLO medium	Pleuropneumonia-like organisms medium
MLST	Multilocus sequence typing
*16S rRNA*	16S ribosomal RNA
