# Supplementary material for: Isolation, Identification, and Molecular Characterization of Mycoplasma bovis from Beef Cattle in Kunming, and Development of a SYBR Green qPCR Assay
Source: Pathogens. 2026 Feb 2;15(2):162. doi: 10.3390/pathogens15020162 (PMC12943494; doi:10.3390/pathogens15020162)
Supplement: Supplementary file 1 [file pathogens-15-00162-s001.zip › pathogens-4074315-supplementary.pdf]

Supplementary

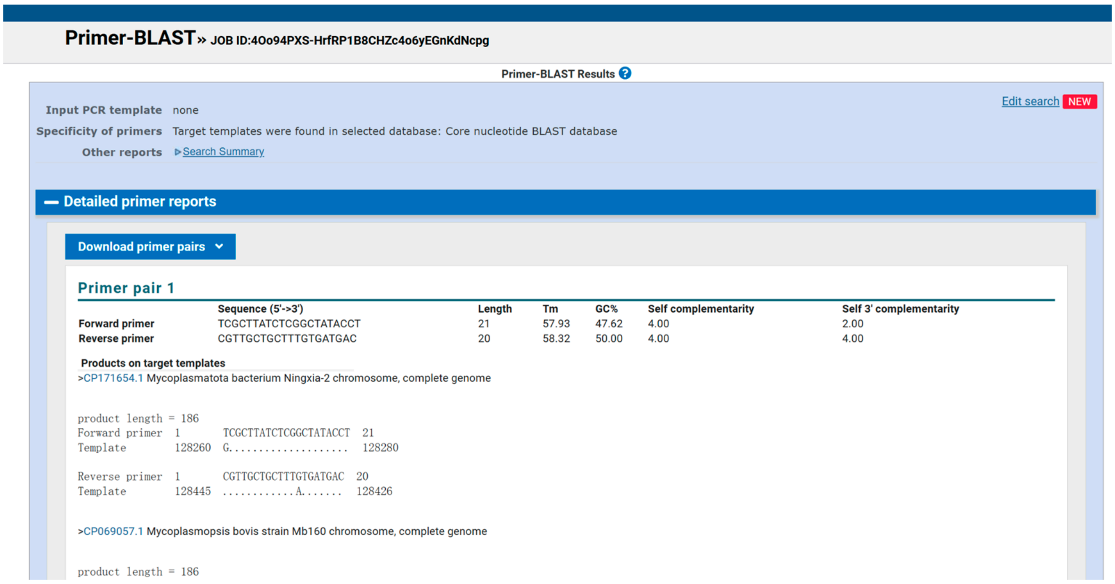

Figure S1. In silico specificity analysis of the *oppD/F* primers using NCBI Primer-BLAST.

Percent Identity (upper triangle); Divergence (lower triangle)

	1	2	3	4	5	6	7	8	9	10	11	12	13	14	15		
1		99.1	99.1	99.1	96.7	99.1	96.7	99.1	80.8	99.1	99.1	99.1	83.2	99.1	79.5	1	Mycoplasmopsis-bovis-strain-XBY01
2	0.0		100.0	100.0	96.7	100.0	96.7	100.0	79.5	100.0	100.0	100.0	82.3	100.0	79.5	2	M.bo-YNXD-A8
3	0.0	0.0		100.0	96.7	100.0	96.7	100.0	79.5	100.0	100.0	100.0	82.3	100.0	79.5	3	Mycoplasma-bovis-CQ-W70
4	0.0	0.0	0.0		96.7	100.0	96.7	100.0	79.5	100.0	100.0	100.0	82.3	100.0	79.5	4	Mycoplasma-bovis-HB0801
5	1.9	1.9	1.9	1.9		96.7	100.0	96.7	91.1	96.7	96.7	96.7	87.6	96.7	89.5	5	Mycoplasma-bovis-isolate-JF4278
6	0.0	0.0	0.0	0.0	1.9		96.7	100.0	79.5	100.0	100.0	100.0	82.3	100.0	79.5	6	Mycoplasma-bovis-strain-NM2012
7	1.9	1.9	1.9	1.9	0.0	1.9		96.7	91.1	96.7	96.7	96.7	87.6	96.7	89.5	7	Mycoplasmopsis-bovis-strain-J279
8	0.0	0.0	0.0	0.0	1.9	0.0	1.9		79.5	100.0	100.0	100.0	82.3	100.0	79.5	8	Mycoplasmopsis-bovis-strain-08M
9	14.0	14.0	14.0	14.0	13.2	14.0	13.2	14.0		79.5	79.5	79.5	83.9	79.5	75.5	9	Mycoplasmopsis-bovis-strain-15DD0261
10	0.0	0.0	0.0	0.0	1.9	0.0	1.9	0.0	14.0		100.0	100.0	82.3	100.0	79.5	10	Mycoplasmopsis-bovis-strain-16M
11	0.0	0.0	0.0	0.0	1.9	0.0	1.9	0.0	14.0	0.0		100.0	82.3	100.0	79.5	11	Mycoplasmopsis-bovis-strain-KRB1
12	0.0	0.0	0.0	0.0	1.9	0.0	1.9	0.0	14.0	0.0	0.0		82.3	100.0	79.5	12	Mycoplasmopsis-bovis-strain-Ningxia-1
13	11.0	11.1	11.1	11.1	8.8	11.1	8.8	11.1	8.5	11.1	11.1	11.1		82.3	77.0	13	Mycoplasmopsis-bovis-strain-PG45.7
14	0.0	0.0	0.0	0.0	1.9	0.0	1.9	0.0	14.0	0.0	0.0	0.0	11.1		79.5	14	Mycoplasmopsis-bovis-strain-Tibet-10
15	2.2	3.6	3.6	3.6	0.4	3.6	0.4	3.6	14.8	3.6	3.6	3.6	10.5	3.6		15	Mycoplasmopsis-bovis-strain-VK24
	1	2	3	4	5	6	7	8	9	10	11	12	13	14	15		

Figure S2. Homology analysis of *VspX* nucleotide sequences.

Percent Identity

Divergence

	1	2	3	4	5	6	7	8	9	10	11	12		
1		100.0	100.0	81.2	100.0	99.5	100.0	99.5	95.5	99.9	99.9	99.8	1	M.bo-YNXD-1
2	0.0		100.0	81.2	100.0	99.5	100.0	99.5	95.5	99.9	99.9	99.8	2	M.bo-YNXD-A1
3	0.0	0.0		81.2	100.0	99.5	100.0	99.5	95.5	99.9	99.9	99.8	3	M.bo-YNXD-A8
4	20.5	20.5	20.5		82.1	81.9	82.1	81.9	82.1	82.1	82.1	81.9	4	Mycoplasma-agalactiae-PG2
5	0.0	0.0	0.0	20.5		99.5	100.0	99.5	95.5	99.9	99.9	99.8	5	Mycoplasma-bovis-CQ-W70
6	0.2	0.2	0.2	20.7	0.2		99.0	99.8	94.6	99.6	99.5	98.8	6	Mycoplasmopsis-bovis-strain-AR3
7	0.0	0.0	0.0	20.5	0.0	0.2		99.5	95.5	99.9	99.9	99.8	7	Mycoplasmopsis-bovis-strain-KRB1
8	0.2	0.2	0.2	20.7	0.2	0.0	0.2		94.8	99.7	99.6	99.0	8	Mycoplasmopsis-bovis-strain-Mb218
9	4.6	4.6	4.6	20.5	4.6	4.8	4.6	4.8		95.5	95.5	95.3	9	Mycoplasmopsis-bovis-strain-NADC51
10	0.1	0.1	0.1	20.5	0.1	0.1	0.1	0.1	4.7		99.9	99.2	10	Mycoplasmopsis-bovis-strain-TO19
11	0.1	0.1	0.1	20.5	0.1	0.1	0.1	0.1	4.7	0.0		99.3	11	Mycoplasmopsis-bovis-strain-VK26
12	0.2	0.2	0.2	20.8	0.2	0.4	0.2	0.4	4.8	0.3	0.3		12	Mycoplasmopsis-bovis-strain-Xinjiang
	1	2	3	4	5	6	7	8	9	10	11	12		

Figure S3. Homology analysis of *p48* nucleotide sequences.

Percent Identity

Divergence

	1	2	3	4	5	6	7	8	9	10	11	12	13		
1		99.0	98.1	99.6	99.6	99.2	99.6	98.7	99.6	99.6	99.4	98.5	99.6	1	M.bo-YNXD-1.seq
2	0.5		97.6	99.2	99.2	98.7	99.2	98.3	99.2	99.2	98.9	98.1	99.2	2	M.bo-YNXD-A1.seq
3	1.7	2.0		97.8	97.8	97.3	97.8	96.9	97.8	97.8	97.5	96.7	97.8	3	M.bo-YNXD-A8.seq
4	0.0	0.5	1.7		100.0	99.5	100.0	98.6	100.0	100.0	99.7	98.6	100.0	4	Mycoplasma-bovis-CQ-W70
5	0.0	0.5	1.7	0.0		99.5	100.0	98.6	100.0	100.0	99.7	98.6	100.0	5	Mycoplasma-bovis-HB0801
6	0.5	0.9	2.2	0.5	0.5		98.3	96.9	98.3	98.3	99.2	97.9	98.3	6	Mycoplasmopsis-bovis-strain AR3-1.seq
7	0.0	0.5	1.7	0.0	0.0	0.5		98.6	100.0	100.0	99.7	98.6	100.0	7	Mycoplasmopsis-bovis-strain-16M
8	0.9	1.4	2.6	1.5	1.5	1.9	1.5		98.6	98.6	98.3	99.5	98.6	8	Mycoplasmopsis-bovis-strain-Mb287
9	0.0	0.5	1.7	0.0	0.0	0.5	0.0	1.5		100.0	99.7	98.6	100.0	9	Mycoplasmopsis-bovis-strain-Millmerran_
10	0.0	0.5	1.7	0.0	0.0	0.5	0.0	1.5	0.0		99.7	98.6	100.0	10	Mycoplasmopsis-bovis-strain-Ningxia-1
11	0.3	0.7	2.0	0.3	0.3	0.7	0.3	1.7	0.3	0.3		97.6	98.4	11	Mycoplasmopsis-bovis-strain-TO-VK-1.seq
12	1.1	1.6	2.9	1.5	1.5	2.0	1.5	0.5	1.5	1.5	2.2		97.2	12	Mycoplasmopsis-bovis-strain-VK12-1.seq
13	0.0	0.5	1.7	0.0	0.0	0.5	0.0	1.5	0.0	0.0	0.3	1.5		13	Mycoplasmopsis-bovis-strain-XBY01
	1	2	3	4	5	6	7	8	9	10	11	12	13		

Figure S4. Homology analysis of *Vpam* nucleotide sequences.

Percent Identity

Divergence

	1	2	3	4	5	6	7	8	9	10	11	12	13	14	15		
1		98.5	100.0	100.0	99.9	100.0	99.7	99.8	100.0	95.3	99.3	95.4	95.4	95.4	95.4	1	Mycoplasmopsis-bovis-strain-XBY01
2	0.8		98.9	98.9	98.8	98.9	98.6	98.7	98.9	94.1	98.1	94.2	94.2	94.2	94.2	2	M.bo-YNXD-A8
3	0.0	0.8		100.0	99.9	100.0	99.7	99.8	100.0	95.3	99.3	95.4	95.4	95.4	95.4	3	Mycoplasma-bovis-CQ-W70
4	0.0	0.8	0.0		99.9	100.0	99.7	99.8	100.0	95.3	99.3	95.4	95.4	95.4	95.4	4	Mycoplasma-bovis-HB0801
5	0.1	0.9	0.1	0.1		99.9	99.6	99.7	99.9	95.2	99.2	95.3	95.3	95.3	95.3	5	Mycoplasma-bovis-strain-NM2012
6	0.0	0.8	0.0	0.0	0.1		99.7	99.8	100.0	95.3	99.3	95.4	95.4	95.4	95.4	6	Mycoplasmopsis-bovis-strain-16M
7	0.3	1.1	0.3	0.3	0.4	0.3		99.8	99.7	95.0	99.0	95.1	95.1	95.1	95.1	7	Mycoplasmopsis-bovis-strain-GJ2F0
8	0.2	1.0	0.2	0.2	0.3	0.2	0.2		99.8	95.1	99.1	95.2	95.2	95.2	95.2	8	Mycoplasmopsis-bovis-strain-GuangXi
9	0.0	0.8	0.0	0.0	0.1	0.0	0.3	0.2		95.3	99.3	95.4	95.4	95.4	95.4	9	Mycoplasmopsis-bovis-strain-KRB1
10	4.9	5.8	4.9	4.9	5.0	4.9	5.2	5.1	4.9		94.5	99.9	99.9	99.9	99.9	10	Mycoplasmopsis-bovis-strain-MJ2
11	0.0	0.9	0.0	0.0	0.1	0.0	0.3	0.2	0.0	4.9		95.3	95.3	95.3	95.3	11	Mycoplasmopsis-bovis-strain-NX114
12	4.8	5.7	4.8	4.8	4.9	4.8	5.1	5.0	4.8	0.1	4.8		99.7	99.9	99.9	12	Mycoplasmopsis-bovis-strain-PG45.9
13	4.8	5.7	4.8	4.8	4.9	4.8	5.1	5.0	4.8	0.1	4.8	0.0		100.0	100.0	13	Mycoplasmopsis-bovis-strain-RM16
14	4.8	5.7	4.8	4.8	4.9	4.8	5.1	5.0	4.8	0.1	4.8	0.0	0.0		99.7	14	Mycoplasmopsis-bovis-strain-TO-VK
15	4.8	5.7	4.8	4.8	4.9	4.8	5.1	5.0	4.8	0.1	4.8	0.0	0.0	0.0		15	Mycoplasmopsis-bovis-strain-VK8
	1	2	3	4	5	6	7	8	9	10	11	12	13	14	15		

Figure S5. Homology analysis of *p81* nucleotide sequences.

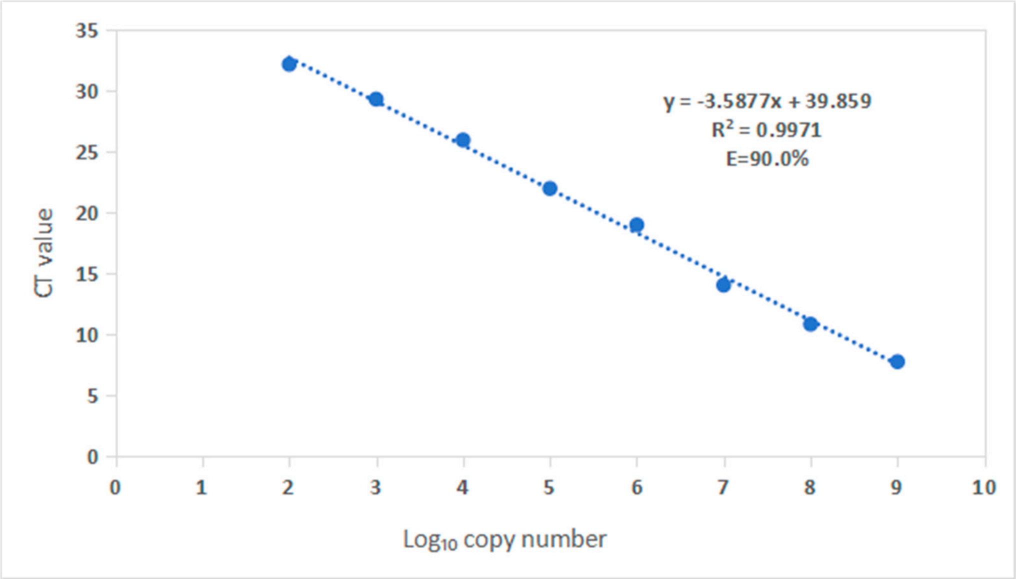

Figure S6. Standard curve of the qPCR assay generated using serial dilutions of the plasmid standard.

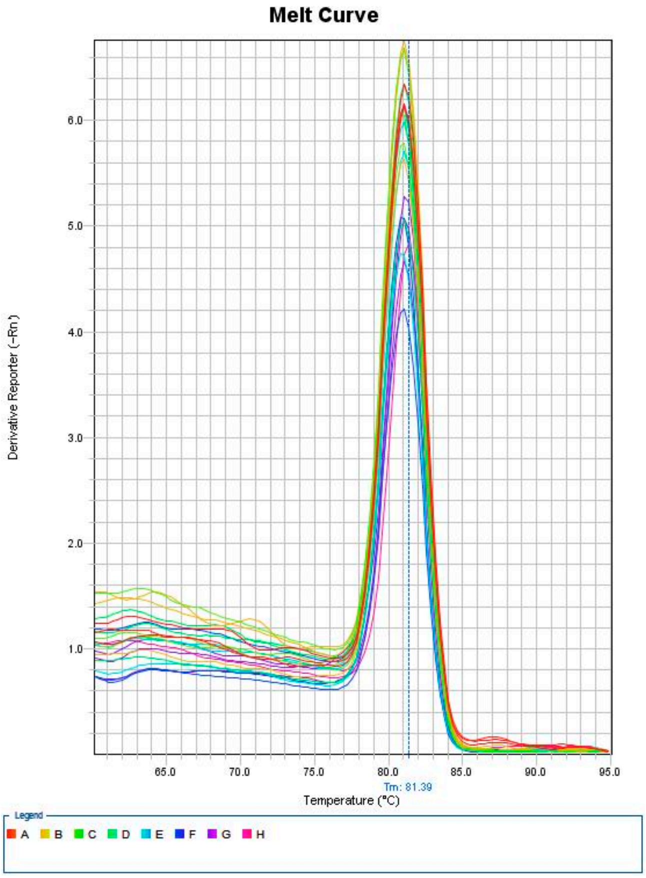

Figure S7. Melting curve analysis showing a single peak, indicating high specificity of the qPCR amplification.

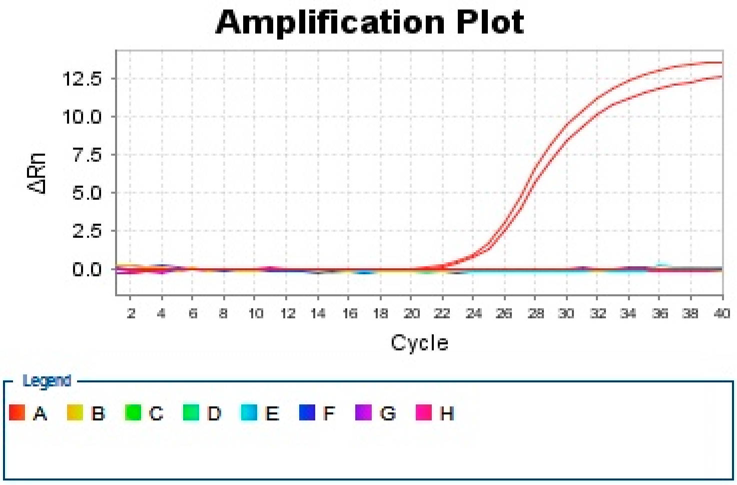

Figure S8. Specificity test results of the assay.

Lane A: positive control plasmid;

Lanes B–H: *Mycoplasma ovipneumoniae* (B), *Salmonella spp.* (C), *Proteus mirabilis* (D), *Akkermansia muciniphila* (E), *Escherichia coli* (F), *Lactobacillus fermentum* (G), and *Enterococcus faecalis* (H).

Table S1. Interpretation criteria for antimicrobial susceptibility of *Mycoplasma bovis.*

Drug class	Drug name	Tablet content (μg/tablet)	Zone of inhibition diameter (mm)		
			S (Susceptible)	I (Intermediate)	R (Resistant)
Macrolides	Erythromycin (E15)	15	≥23	14~22	≤13
Quinolones	Ciprofloxacin (CIP)	5	≥26	22~25	≤21
	Enrofloxacin (ENR)	5	≥21	16~20	≤15
	Norfloxacin (NOR)	10	≥17	13~16	≤12
Lincosamides	Lincomycin (LC)	2	≥21	15~20	≤14
	Clindamycin (CC)	2	≥23	15~22	≤14
Tetracyclines	Doxycycline (DX)	30	≥14	11~13	≤10
	Tetracycline (TE)	30	≥15	12~14	≤11
Aminoglycosides	Streptomycin (SM)	10	≥18	12~14	≤11
	Gentamicin (GM)	10	≥18	15~17	≤14
	Spectinomycin	10/30	≥15/18	13~14;	≤12/≤14
	Amikacin (AK)	30	≥20	17~19	≤16
Nitrofurans	Nitrofurantoin (FN)	300	≥17	15~16	≤14

Table S2. Primers information for MLST housekeeping genes.

Gene	Primer sequence	Product	Annealing
dnaA	F: TCAAATCGTGAAGTCGACAA<br>R: CTTCCCAATTTGTTCCAGTG	924bp	55℃
tkt	F: CATGTGAATATAAATGCACTGC<br>R: TGATAAGGCAATTACTGAAGC	677bp	55℃
gltX	F: TGGTGAGTATTCAATAAGGT<br>R: GTTTTGAGAATCATTGCA	530bp	47℃
gpsA	F: AAAATGTGAGGAATTGATCA<br>R: CCAATTCCAATTGTAAAAC	521bp	47℃
gyrB	F: AGCTTGCTAATTGCACCA<br>R: TATTTTGAACAAATTTTGCAT	678bp	47℃
pta2	F: AATTCGTAATGGCAAAGAAG<br>R: CTTAGCTTTTCTTACATTTAGGT	490bp	55℃
tdk	F: ATGTATTTAAAAAGTGGATTAGG<br>R: TATCTCATAGCTTTTTTAGC	572bp	47℃

Table S3. Primers for detection of virulence genes.

Gene	Primer sequence (5′–3′)	Product	Annealing
vspY2	F: TTTTATTGTTAGGTTCTCTTAC<br>R: TCTTGGAAATATTTTTTGTTG	777 bp	46℃
VspX	F: TCAATAAATTGCTAATATCTGCTG<br>R: TCAAATATTGGTCTAAGATCAGC	665 bp	49℃
VspHB0801-1	F: GCTTGGATCAGTAGCTTCATTGGC<br>R: TTATTTTTTGCCTGATTCAGCTTC	628 bp	55℃
Vpma	F: TCACTTGGTTCACTATCTATTTT<br>R: TAAGTTTTATTTCACCATCAAGTA	1331 bp	48℃

p48	F: ATTCTATCTATTTTTAGGAGCCGC<br>R: TTGTGTTTCTTTAGCCAATCAGTT	1387 bp	52℃
p81	F: CTATCCCGCTTTTAGCAGCAGTGT<br>R: TTTATCGAAATCTTCTAGTGGTTG	2096 bp	55℃

S2.9 Detailed Experimental Procedures

S2.9.1 DNA Extraction and Primer Design

Genomic DNA was extracted from standard Mycoplasma bovis cultures and clinical samples using commercially available DNA extraction kits specific for mycoplasmas or Gram-negative bacteria, according to the manufacturers' instructions. Based on multiple M. bovis *oppD/F* gene sequences retrieved from GenBank, a specific primer pair was designed using Primer Premier 5.0 software to generate an amplicon of 186 bp. Primer specificity was subsequently evaluated in silico using NCBI Primer-BLAST against the nucleotide (nt) database, confirming exclusive amplification of M. bovis with no predicted amplification in non-target species (Figure S1).

S2.9.2 Construction of the Positive Plasmid Standard

S2.9.2.1 Amplification of the Plasmid Standard

PCR amplification was performed using DNA from three *M. bovis* isolates as templates in a 25 μL reaction system. The cycling conditions were as follows: pre-denaturation at 94 °C for 6 min; 35 cycles of denaturation at 94 °C for 30 s, annealing at 56 °C for 30 s, and extension at 72 °C for 35 s; followed by a final extension at 72 °C for 8 min. PCR products were confirmed by electrophoresis on a 1% agarose gel and subsequently purified.

S2.9.2.2 Plasmid Cloning and Sequence Verification

The purified PCR product was ligated into the pMD19-T vector and transformed into *E. coli* DH5α competent cells (Dalian TaKara Biotechnology Co., Ltd.). Positive clones were screened by PCR and 1% agarose gel electrophoresis. Verified clones were sequenced, and the obtained sequences were compared with those in the NCBI

GenBank database to confirm the absence of mutations. The validated plasmid was designated as pMD19-T-oppD/F186.

S2.9.3 qPCR Reaction System and Optimisation

A recombinant plasmid containing 1.0×10^5 copies/μL was used as the template. The reaction conditions, including primer concentration (0.6–10 μM), annealing temperature (57–62 °C), and template volume (0.5–3 μL), were optimized using a single-factor approach to determine the optimal parameters for qPCR amplification.

S2.9.4 Establishment of Standard and Melting Curves

The standard plasmid (1.0×10^9 copies/μL) was serially diluted 10-fold to generate concentrations ranging from 1.0×10^9 to 1.0×10^1 copies/μL, with three replicates for each concentration. SYBR Green I-based qPCR was performed to construct the standard curve and calculate amplification efficiency. Each 20 μL reaction contained 10 μL of 2× SYBR® Green Pro Taq HS Premix (Rox Plus) (Hunan Accurate Biotechnology Co., Ltd.), 0.4 μL of each primer, 2 μL of template DNA, and nuclease-free water to volume. The cycling program consisted of an initial denaturation at 95 °C for 30 s, followed by 40 cycles of 95 °C for 5 s and 60 °C for 30 s, ending with melting curve analysis.

S2.9.5 Specificity Analysis

The optimized SYBR Green I qPCR assay was evaluated for cross-reactivity using non-target DNA from *Mycoplasma ovipneumoniae*, *Salmonella spp.*, *Proteus mirabilis*, *Akkermansia muciniphila*, *Escherichia coli*, *Lactobacillus fermentum*, *Mannheimia haemolytica*, and *Enterococcus faecium*. Recombinant plasmid DNA served as the positive control, and ddH_2O was used as the negative control.

S2.9.6 Sensitivity Analysis

10-fold serial dilutions of the standard plasmid, ranging from 1.0×10^9 to 1.0×10^1 copies/μL, were used to determine the limit of detection (LOD) of the assay.

S2.9.7 Repeatability and Stability Evaluation

Five plasmid concentrations (1.0×10^9 to 1.0×10^5 copies/μL) were tested in triplicate across three independent batches. The coefficient of variation (CV) of the Ct values was calculated to assess assay repeatability and stability.

S2.9.8 Detection and Validation with Clinical Samples

A total of 58 bovine samples collected from farms in Kunming, Yunnan Province, were tested using both the SYBR Green qPCR assay developed in this study and a conventional PCR method. The concordance rate and methodological consistency between the two assays were evaluated.
